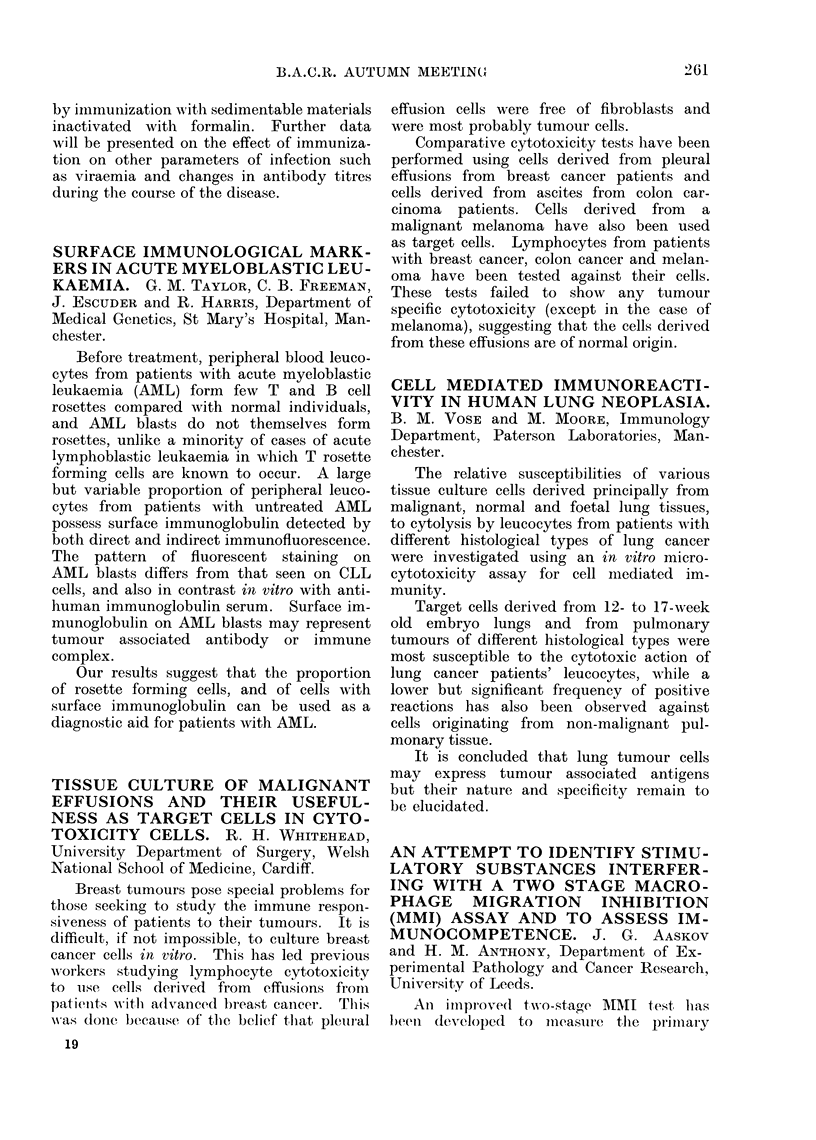# Proceedings: Cell mediated immunoreactivity in human lung neoplasia.

**DOI:** 10.1038/bjc.1975.40

**Published:** 1975-02

**Authors:** B. M. Vose, M. Moore


**TISSUE CULTURE OF MALIGNANT EFFUSIONS AND THEIR USEFULNESS AS TARGET CELLS IN CYTOTOXICITY CELLS.** R. H. WHITEHEAD, University Department of Surgery, Welsh National School of Medicine, Cardiff.

Breast tumours pose special problems for those seeking to study the immune responsiveness of patients to their tumours. It is difficult, if not impossible, to culture breast cancer cells *in vitro*. This has led previous workers studying lymphocyte cytotoxicity to use cells derived from effusions from patients with advanced breast cancer. This was done because of the belief that pleural effusion cells were free of fibroblasts and were most probably tumour cells.

Comparative cytotoxicity tests have been performed using cells derived from pleural effusions from breast cancer patients and cells derived from ascites from colon carcinoma patients. Cells derived from a malignant melanoma have also been used as target cells. Lymphocytes from patients with breast cancer, colon cancer and melanoma have been tested against their cells. These tests failed to show any tumour specific cytotoxicity (except in the case of melanoma), suggesting that the cells derived from these effusions are of normal origin.